# Statistical distractor learning modulates perceptual sensitivity

**DOI:** 10.1167/jov.21.12.3

**Published:** 2021-11-05

**Authors:** Dirk van Moorselaar, Jan Theeuwes

**Affiliations:** 1 Department of Experimental and Applied Psychology, Vrije Universiteit, Amsterdam, the Netherlands; 2 Institute of Brain and Behaviour, Amsterdam, the Netherlands; 3 Department of Experimental and Applied Psychology, Vrije Universiteit, Amsterdam, the Netherlands; 4 Institute of Brain and Behaviour, Amsterdam, the Netherlands

**Keywords:** statistical learning, suppression, attention, visual search

## Abstract

The present study used perceptual sensitivity (*d*′) to determine the spatial distribution of attention in displays in which participants have learned to suppress a location that is most likely to contain a distractor. Participants had to indicate whether a horizontal or a vertical line, which was shown only briefly before it was masked, was present within a target shape. Critically, the target shape could be accompanied by a singleton distractor color, which when present appeared with a high probability at one display location. The results show that perceptual sensitivity was reduced for locations likely to contain a distractor, as *d*′ was lower for this location than for all other locations in the display. We also found that the presence of an irrelevant color singleton reduced the gain for input at the target location, particularly when the irrelevant singleton was close to the target singleton. We conclude that, through the repeated encounter with a distractor at a particular location, the weights within the attentional priority map are changed such that the perceptual sensitivity for objects presented at that location is reduced relative to all other locations. This reduction of perceptual sensitivity signifies that this location competes less for attention than all other locations.

## Introduction

When we search the environment for relevant information, we are constantly trying to minimize the interference caused by visual distractors. Such distractors appear especially difficult to ignore when their features stand out. A continuous debate in the attention literature centers around the question of whether salient stimuli involuntarily attract attention or whether such automatic attentional capture can be avoided (Luck, Gaspelin, Folk, Remington, & Theeuwes, 2021). Although there continue to be opposing viewpoints in this debate, there is a growing consensus that human observers, often implicitly, learn from regularities in the environment that can reduce the interference caused by salient distractors (Chelazzi, Marini, Pascucci, & Turatto, 2019; Theeuwes, 2019; van Moorselaar & Slagter, 2020). For example, there are now many reports of observers being sensitive to imbalances in the spatial distribution of salient distractors such that distractors are more efficiently ignored when presented more often at one location than at all other locations (Di Caro, Theeuwes, & Della Libera, 2019; Ferrante, Patacca, Di Caro, Della Libera, Santandrea, & Chelazzi, 2018; van Moorselaar, Daneshtalab, & Slagter, 2021; Wang & Theeuwes, 2018b; Wang & Theeuwes, 2018c).

In a series of experiments, Theeuwes and colleagues (Failing, Wang, & Theeuwes, 2019; Wang & Theeuwes, 2018b; Wang & Theeuwes, 2018c) employed the classic additional singleton task and showed that statistical regularities regarding the location of the salient distractor affected attentional selection. Critically, participants responded faster and more accurately to the target singleton when the salient distractor singleton was presented at a high-probability location relative to the other, low-probability locations. This finding was interpreted as evidence that, when presented at the high-probability location, the color singleton distractor caused less attention capture than when presented at any of the regular locations. Also, if the target happened to be presented at the location that was most likely to contain a distractor, participants were relatively slow in responding to the target. On the basis of these studies, it was concluded that, through statistical learning, relative to all other locations the location that is likely to contain a distractor becomes suppressed (Ferrante et al., 2018; Wang & Theeuwes, 2018b; Wang & Theeuwes, 2018c; Zhang, Allenmark, Liesefeld, Shi, & Muller, 2019). It should be noted that this learned suppression is instantiated relatively quickly and then remains stable as long as the spatial imbalance is incorporated across visual searches (Lin, Li, Wang, & Theeuwes, 2021).

Unlike the notion that distractor suppression is usually *reactive*, resulting from (rapidly) disengaging attention from an attended location (Moher & Egeth, 2012; Theeuwes, 2010), it is more likely that, when statistical learning is involved, suppression is *proactive*; that is, the high-probability distractor location is already suppressed prior to the onset of the search display (Huang, Vilotijević, Theeuwes, & Donk, 2021; Wang, van Driel, Ort, & Theeuwes, 2019). Although virtually all studies up to now have assumed that learned spatial suppression operates on a pre-selective stage of priority computation (e.g., Ferrante et al., 2018; Sauter, Liesefeld, & Müller, 2019; Sauter, Liesefeld, Zehetleitner, & Müller, 2018; Wang & Theeuwes, 2018b; Wang & Theeuwes, 2018c), it should be noted that the vast majority of these studies relied on response time (RT) measures, which critically do not allow for a separation between perceptual and decision-level effects of attention. Indeed, the suppression of the high-probability distractor location is inferred on the basis of RTs to the target singleton, which are faster when a distractor is presented at this high-probability location and critically slower when a target happens to be presented at this location.

Even though these RT measures are compelling, reduced perceptual sensitivity for signals presented at the high-probability location relative to all other locations would provide unequivocal evidence in support of suppression. Therefore, in this study we used measures derived from signal detection theory (SDT) to determine perceptual sensitivity (*d*′), which is assumed to represent the sensitivity of the system to the occurrence of a signal, expressed in signal-to-noise ratio units (Green & Swets, 1966; Macmillan & Creelman, 2004). If the sensitivity at the high-probability location is reduced, it would provide direct evidence for a mechanism of perceptual suppression unrelated to effects that operate at post-selective stages, such as quicker attentional disengagement at high-probability distractor locations and slower disengaging from distractors at low-probability locations (Sauter, Hanning, Liesefeld, & Müller, 2020).

Using a signal detection version of the additional singleton task, Theeuwes, Kramer, and Kingstone (2004) (see also Theeuwes & Chen, 2005) showed lower visual sensitivity (*d*′) on distractor-present displays, indicating that the singleton distractor cost could not be attributed to operations occurring after initial selection but rather modulated target detectability by reducing the gain for inputs at the target location. Moreover, it was shown that this effect was driven by distractors in close proximity to the target, as distractors farther away from the target did not affect target detectability (*d*′). Therefore, here we not only investigated whether statistical distractor learning modulated target detectability but also examined the statistical learning effect as a function of the distance between targets and distractors.

For this purpose, in an online study, we employed a method similar to that used by Theeuwes et al. (2004). Participants were required to make a two-choice forced decision regarding the presence of a specific orientation of a target bar in displays with and without a colored distractor. Critically, when present, this colored singleton appeared with a higher probability at one specific location. If statistical distractor learning indeed operates on a pre-selective stage of priority computation, we would expect visual sensitivity (*d*′) to be lower when a target happens to appear at the high-probability distractor location, and we would expect sensitivity to be higher for distractors at high-probability relative to low-probability locations.

## Methods

### Participants

Data collection continued until we collected 48 complete datasets (i.e., datasets where the experiment was aborted before completion were ignored), a sample size that was based on an a priori power analysis (α = 0.05, power = 0.95) based on the main effect of distractor presence as reported in Theeuwes et al. (2004). The final sample contained 48 first-year students (40 female; mean age = 21.1 years; range, 17–40) who participated for research credits. One dataset was excluded because the participant performed the experiment twice, and three subjects were replaced because average accuracy across conditions was more than 2.5 *SD* below the grand mean. The ethical committee of the Faculty of Behavioral and Movement Sciences at Vrije Universiteit approved the study, which conformed to the tenets of the Declaration of Helsinki, and participants provided digital informed consent via Qualtrics (Qualtrics, Provo, UT) prior to participation.

### Task, stimuli, and procedure

As the experiment was conducted online, and we thus had no control over the experimental setting, for replication purposes we report pixels and RGB values to describe the stimuli. The experiment was created in OpenSesame 3 (Mathôt, Schreij, & Theeuwes, 2012) using OSWEB (versions 1.3.11 and 1.3.13^1^) and run using JATOS (Lange, Kühn, & Filevich, 2015) on desktop computers or laptops.

**Figure 1.**
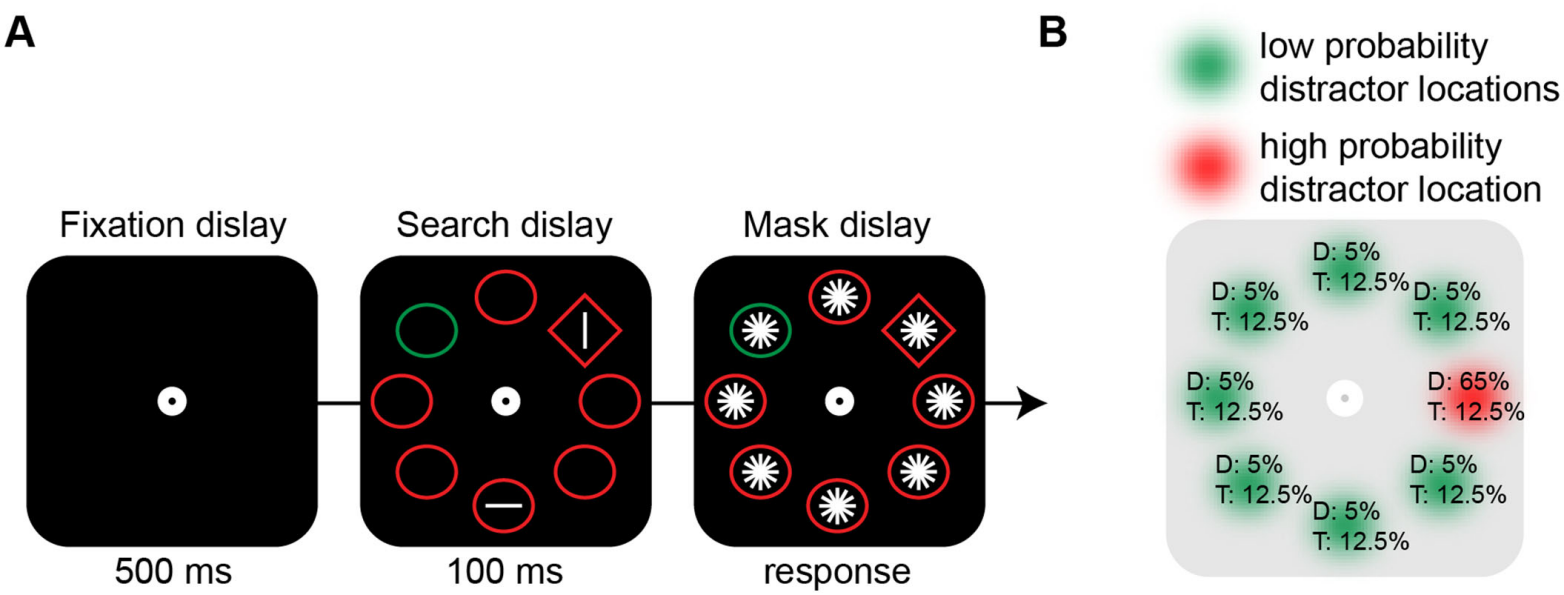
Experimental paradigm. (A) Graphical illustration of the sequence and timing of stimulus events presented on each trial. For each display participants had to indicate whether a given orientation (e.g., horizontal) was present within the unique shape singleton (in this case, a diamond). The singleton distractor color, when present, was more likely to appear in one location along the imaginary circle. (B) Schematic representation of the spatial regularities of the distractor. Percentages at each location represent the probabilities of the distractor (D) and the target (T) appearing at a given location.

Each trial started with a 500-ms fixation display that consisted of a white circle on a black background. Subsequently, a 100-ms search display appeared with eight equidistant shapes in a circular configuration around fixation (radius, 224 pixels). Each display contained a circle (radius, 45 pixels) among diamonds (100 × 100 pixels), or vice versa, each with a red (255/0/0) or a green (0/146/69) outline on a black background. On distractor-present trials (66.7%), the outline of one of the homogeneous shapes had a different color than the other stimuli in the display (i.e., red or green). Critically, when present, this singleton distractor appeared with a higher probability (65%) at one of the eight locations.^2^ Collapsed across distractor-present and distractor-absent trials, targets appeared equally often on all locations (Figure 1B). A horizontal or vertical white (255/255/255) target bar (counterbalanced across trials) was consistently placed inside the target shape singleton (i.e., unique shape), whereas another horizontal or vertical bar (selected at random) appeared at random in one of the neutral stimuli. That is, the non-target line never appeared inside the singleton distractor. Finally, a masked display, in which a set of six white line segments (60 pixels; 0°–180° in steps of 30°) were placed at the center of each stimulus, was shown until response.

Participants were instructed to keep their eyes at fixation and to indicate via button press whether the target orientation (i.e., horizontal or vertical; counterbalanced across participants) was present (press “P”) or absent (press “A”) inside the target shape. The line length (start value, 40 pixels; minimum length, 2 pixels; maximum length, 60 pixels) of the lines inside the search display was adjusted every four trials based on average accuracy in the preceding 16 trials: +4 pixels for accuracy below 65% (i.e., six or more errors); +2 pixels for accuracy below 70% (i.e., five errors); −2 pixels for accuracy above 80% (i.e., three errors); and −4 pixels for accuracy above 85% (i.e., two or fewer errors). Doing so ensured that performance remained near 75% correct. In case of an incorrect response, the fixation circle turned into a cross for 495 ms, whereas it remained white for 250 ms in case of a correct response.

Participants were encouraged to respond as accurately as possible and completed seven blocks of 120 trials each (trial order randomized), which were preceded by a series of 20 practice trials. The practice block, in which the line length was not adjusted, continued to repeat until average accuracy was above 66%. Halfway through each block, participants were given the opportunity for a short break, and at the end of each block they received feedback on their performance (i.e., mean accuracy and mean response time), and they were encouraged to take a break. After the last block, participants were first asked whether they noticed that one location had a higher distractor probability. Subsequently, a display with white circles, each with a unique identifier, corresponding to one of the search locations was shown, and participants had to indicate (and, if necessary, guess) which location they believed contained the singleton distractor most frequently throughout the experiment.

### Data analysis

Visual search performance was measured using signal detection theory (Green & Swets, 1966; Macmillan & Creelman, 2004), where the sensitivity index (*d*′) measures the discriminability between a target signal (i.e., target orientation) and noise (i.e., the non-target orientation). The *d*′ was calculated for each participant per condition of interest making use of the log-linear approach to control for extreme rates (Hautus, 1995) after excluding responses shorter than 200 ms and longer than 2000 ms (4.8%). The Z-transformed probabilities of reporting the target orientation as being present when it was indeed present (hit) and when it was actually absent (false alarm) were calculated using the norm.ppf function in scipy (Virtanen, Gommers, Oliphant, Haberland, Reddy, Cournapeau, & Bright, 2020).

### Statistics

Calculated *d*′ values were analyzed with repeated-measures ANOVAs, where reported *p* values were Greenhouse–Geiser corrected in case of sphericity violations, followed by planned comparisons with paired *t*-tests using JASP software (JASP Team, 2018).

## Results and discussion

Overall, performance was at 70.6% correct, indicating that at least for a subset of participants the staircase procedure resulted in ∼5% lower accuracy than intended, arguably because line length (*M* = 41.5; range, 13.5–58.9) was virtually at the ceiling (>53.0) for a small subset of participants (*n* = 8). Critically, however, as the same staircase procedure was used across conditions, and conditions varied randomly across trials, condition differences cannot be explained by systematic differences across conditions resulting from the staircase procedure. Before examining *d*′ across conditions, we first analyzed averaged response times to exclude any alternative explanation in terms of a speed–accuracy trade-offs.^3^ Critically, distractors were more efficiently ignored at high-probability relative to low-probability locations, *t*(47) = 2.9, *p* = 0.005, *d* = 0.43, demonstrating that reduced sensitivity at the high-probability distractor location could not be attributed to a speed–accuracy trade-off (Table 1).

**Table 1.** Mean response times and standard deviations across distractor conditions.

Distractor condition	*M*	*SD*
Absent	646.9	171.1
High-probability location	652.4	178.0
Low-probability location	664.1	185.0

### Reduced sensitivity at the high-probability distractor location

**Figure 2.**
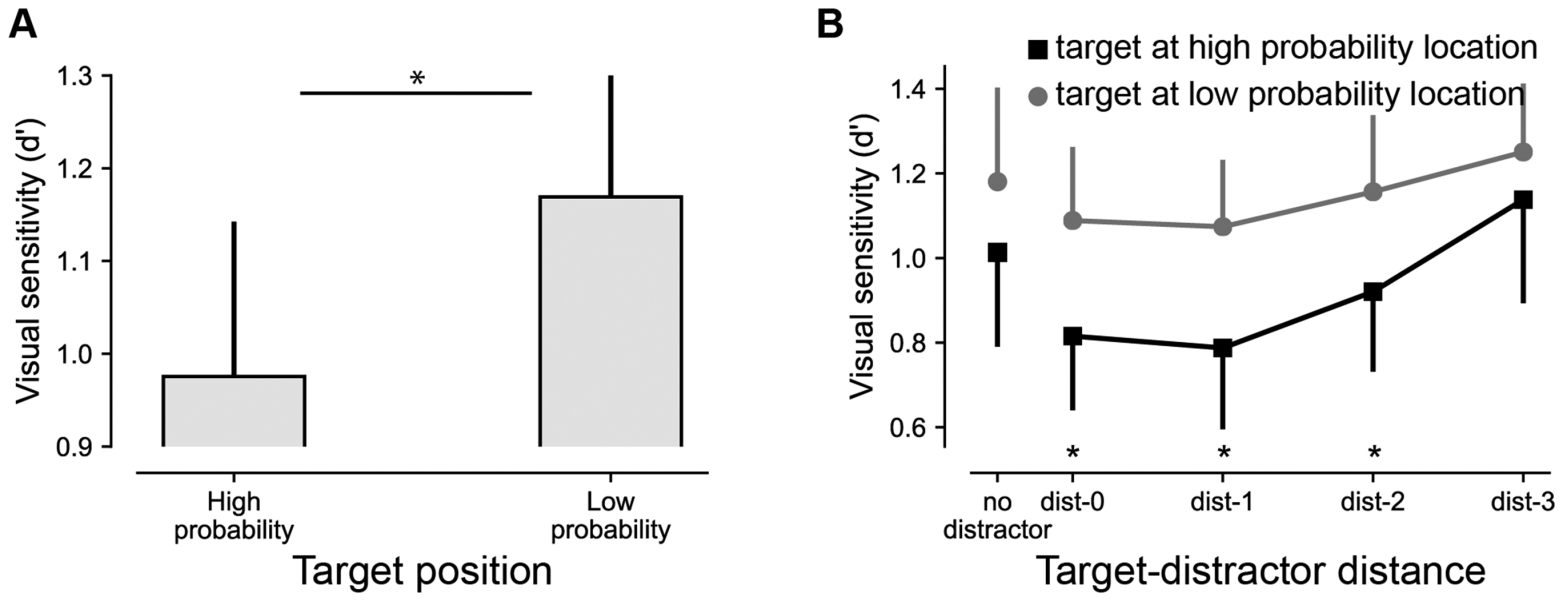
Statistical learning results in reduced sensitivity for targets at high-probability distractor locations. (A) *d*′ as a function of target position. (B) *d*′ for targets at high-probability (black) and low-probability (gray) distractor locations as a function of distance (measures as the number of elements between singletons) between targets and distractors. In all analyses, displays with distractors at high-probability distractor locations were excluded. All error bars here and in subsequent plots represent 95% within-subject confidence intervals (Morey, 2008).

To establish attentional suppression (i.e., reduced perceptual sensitivity) at the high-probability location, we contrasted *d*′ for targets at low- and high-probability distractor locations (Figure 2A). In doing so, we made sure that all trials with a distractor at the high-probability distractor location were excluded from the analysis, such that any observed differences could not be explained by reduced distractor interference at that location (van Moorselaar, Lampers, Cordesius, & Slagter, 2020). A planned comparison confirmed that signal sensitivity was reliably lower at high-probability relative to low-probability distractor locations, *t*(47) = 2.3, *p* = 0.024, *d* = 0.34, consistent with the notion that the high-probability distractor location was suppressed. Counter to the typical additional singleton paradigm, here only two out eight stimuli had an embedded line figure, which may have resulted in relatively large compatibility effects (Schreij, Owens, & Theeuwes, 2008), with overall responses being fastest when the line orientations within the two stimuli were compatible. Although this was indeed the case, a control analysis showed that compatibility did not interact with target position, nor did distance between the line elements modulate the effect of interest (for a detailed analysis, see Supplementary Material SA).

### Target sensitivity increases with increasing target–distractor distance

Following the finding by Theeuwes et al. (2004) that target detectability (*d*′) was selectively affected by distractors in close proximity to the target, we further examined how the reduced sensitivity at the high-probability distractor location was influenced by distractors that were in close proximity to the target compared with those that were farther away. For this purpose, after again excluding all trials with distractors at high-probability locations, we entered *d*′ estimates into a repeated-measures ANOVA with within-subject factors of target position (high-probability location, low-probability location) and target–distractor distance (0, 1, 2, 3), where distance was quantified by the number of elements between singletons. As visualized in Figure 2B, it appeared that reduced signal sensitivity at high-probability distractor locations, with the main effect of target position, *F*(1, 47) = 8.8, *p* = 0.005, $n_{p}^{2}$ = 0.16, was most pronounced when distractors appeared in close proximity to the target. Critically, however, this was not supported by a reliable interaction, *F*(3, 141) = 0.6, *p* = 0.63, $n_{p}^{2}$ = 0.012, *BF_01_* = 22.4, and a Bayesian analysis showed that the absence of an interaction was 22 times more likely than suppression at the high-probability location being modulated by target–distractor distance. Nevertheless, we interpret this null finding with caution, given that the experiment was not specifically designed to examine this effect, resulting in a relatively low number of observations per cell. As also visualized in Figure 2B, the main effect of target position was, however, accompanied by a main effect of distance, *F*(3, 141) = 3.2, *p* = 0.024, $n_{p}^{2}$ = 0.064, which was characterized by a linear trend, *t*(141) = 3.5, *p* < 0.001 (collapsed over all target positions). Replicating Theeuwes et al. (2004), relative to distractor-absent displays distractors in close proximity to the target reduced target detecttability, *t*(47) = 2.6, *p* = 0.012, *d* = 0.38 (95% confidence interval [CI], 0.04–0.28) for distance 0; *t*(47) = 2.9, *p* = 0.005, *d* = 0.42 (95% CI, 0.05–0.27) for distance 1 (collapsed over all target positions). This was not the case for more distant distractors, *t*(47) = 1.3, *p* = 0.19, *d* = 0.19 (95% CI, −0.05 to 0.23), *BF_01_* = 2.8 for distance 2; *t*(47) = −1.9, *p* = 0.064, *d* = −0.27 (95% CI, −0.32 to 0.00), *BF_01_* = 1.2 for distance 3 (collapsed over all target positions).

### Suppressed distractors increase target sensitivity

**Figure 3.**
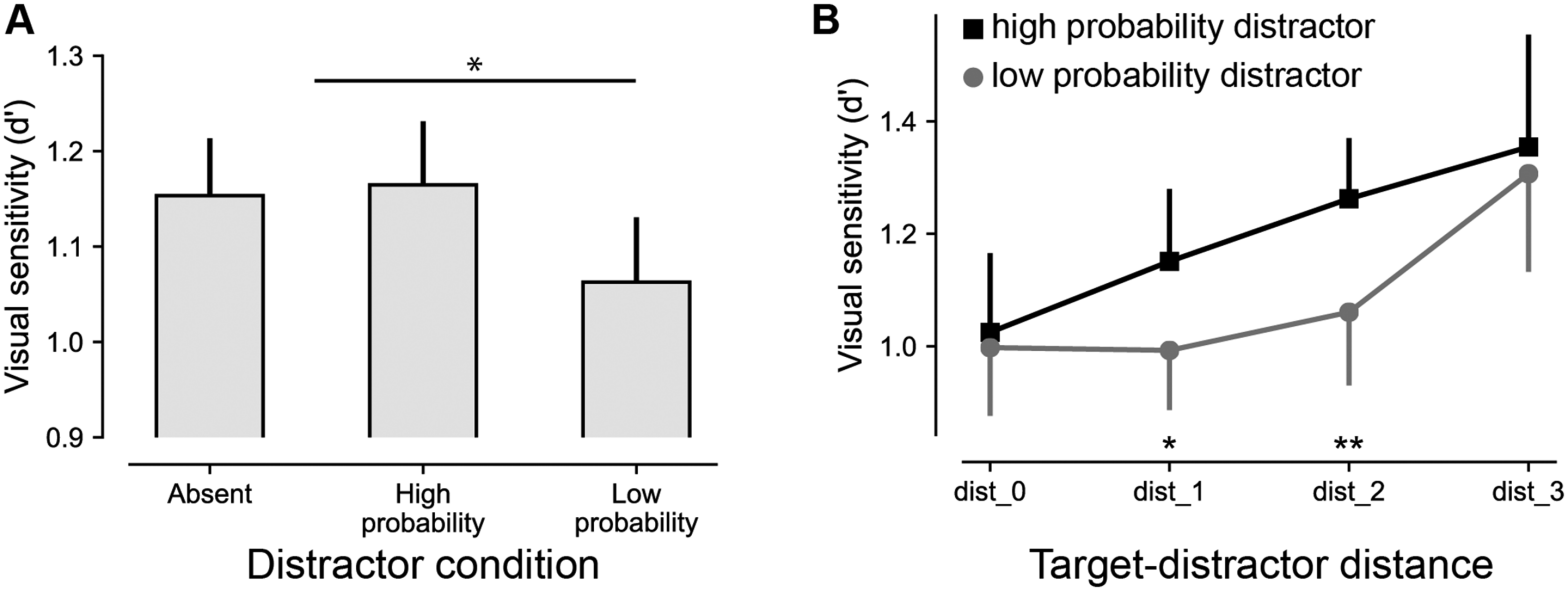
Learned distractor suppression increases target sensitivity. (A) *d*′ as a function of distractor condition. (B) *d*′ for distractors at high-probability (black) and low-probability (gray) distractor locations as a function of distance (measures as the number of elements between singletons) between targets and distractors. Please note that matching conditions in Figures 2B and 3B are not exactly overlapping given that the gray line in panel B contains targets at both high- and low-probability distractor locations, whereas this is not the case in Figure 2B.

In the previous analyses we intentionally excluded all trials with distractors at high-probability distractor locations to examine how statistical learning modulated sensitivity at the target location. Next, we investigated distractor costs as a function of distractor position. As visualized in Figure 3A and shown in Table 2, *d*′ was higher on distractor-absent than on distractor-present trials, but critically only for distractors at low-probability locations. This was confirmed by a repeated-measures ANOVA with a within-subject factor of distractor condition (high-probability location, low-probability location, absent), which yielded a main effect, *F*(1, 47) = 4.5, *p* = 0.013, $n_{p}^{2}$ = 0.088. Subsequent planned pairwise comparisons confirmed that *d*′ was reliably reduced relatively to distractor absent trials on low-probability, *t*(47) = 2.5, *p* = 0.015, *d* = 0.36, but critically not at high-probability distractor locations, *t*(47) = 0.3, *p* = 0.78, *d* = 0.04, *BF_01_* = 6.1. Moreover, there also was a reliable difference between *d*′ at high- and low-probability locations, *t*(47) = 2.6, *p* = 0.014, *d* = 0.37, confirming that the high-probability distractor location was suppressed. Also, in line with the target-tuned analysis, although distractors close to the target again resulted in more distractor interference with a main effect of target–distractor distance, *F*(2, 115) = 9.0, *p* < 0.001, $n_{p}^{2}$ = 0.071, this target–distractor distance effect did not interact, *F*(2, 106) = 0.7, *p* = 0.50, $n_{p}^{2}$ = 0.007, *BF_01_* = 14.0, with the observed benefit for distractors at the high-probability distractor location with a main effect of distractor position, *F*(1, 47) = 6.4, *p* = 0.015, $n_{p}^{2}$ = 0.015.

**Table 2.** Mean *d*′, hit rate, and false alarm rate per distractor condition and target position as calculated with the log-linear approach (Hautus, 1995).

	*d*′		Hit rate		False alarm rate	
	*M*	*SD*	*M*	*SD*	*M*	*SD*
Distractor condition						
No distractor	1.15	0.27	0.73	0.07	0.30	0.06
High-probability distractor	1.16	0.23	0.74	0.07	0.30	0.06
Low-probability distractor	1.06	0.25	0.73	0.08	0.33	0.08
Target position						
High-probability location	0.98	0.54	0.70	0.11	0.34	0.10
Low-probability location	1.17	0.26	0.74	0.07	0.30	0.07

A control analysis, in which we excluded all trials in which the distractor location repeated (18.8%), replicated the main effect of distractor position as shown in Figure 3A, *F*(1, 47) = 4.0, *p* = 0.021, $n_{p}^{2}$ = 0.079, with sensitivity again being significantly higher at high-probability relative to low-probability distractor locations, *t*(47) = 2.4, *p* = 0.023, *d* = 0.34, indicating that intertrial priming effects could not account for the observed suppression (Maljkovic & Nakayama, 1994).

### Awareness of the high-probability distractor location

Finally, we examined whether participants noticed that distractors appeared with higher probability at a given location. Although 13 subjects indicated that they noticed the spatial imbalance of distractor locations, only two of them actually indicated the correct high-probability distractor location (in total, 10 subjects indicated the correct location). Also, the overall pattern of results was the same irrespective of whether the analyses were limited to subjects who did or did not correctly indicate the high-probability distractor location. This suggests that the observed effects largely reflect implicit statistical learning rather than a deliberate strategy resulting in an overt shift of attention away from the high-probability distractor location. Indeed, exploratory control analyses indicated that reported results reflected learned suppression of the high probability location, rather than secondary suppression stemming from a strategic overt shift of attention away from this location (Supplementary Material SB; see also van Moorselaar et al., 2021; Wang & Theeuwes, 2018a; Wang & Theeuwes, 2018b; Wang & Theeuwes, 2018c) for studies that did monitor eye movements).

## General discussion

The present study shows that statistical regularities regarding the location of the distractor influence visual selection via suppression of locations that frequently contain a distractor. Previous studies, largely relying on RT measures, have shown that such regularities affect both attentional capture by the distractor and efficiency of target selection (e.g., Failing et al., 2019; Wang & Theeuwes, 2018b; Wang & Theeuwes, 2018c). Based on these findings, it is argued that repeated encounters with a distractor at a given location cause lingering biases on a spatial priority map such that that location competes less for attention. Instead of claiming that suppression is *reactive* (Moher & Egeth, 2012; Theeuwes, 2010), due to statistical learning suppression becomes *proactive* (Huang et al., 2021; Wang et al., 2019). Here, we provide unequivocal evidence for the idea that the high-probability distractor location is suppressed by showing reduced perceptual sensitivity for signals presented at the high-probability location relative to all other locations. At the same time, our results demonstrate that on top of the effect of statistical learning there was an effect of display configuration, with target sensitivity being lowest in displays with nearby distractors.

Counter to previous studies here we relied on a metric (*d*′) derived from SDT (Green & Swets, 1966; Macmillan & Creelman, 2004), which is thought to solely reflect perceptual effects of attention and hence, unlike RT measures, is insensitive to effects originating at post-selective stages. Consequently, the finding that *d*′ is reduced in displays with targets at high-probability distractor locations and is increased in displays with distractors at that location cannot be explained by post-capture processes. As a result of statistical learning, which occurs with little, if any explicit knowledge, weights within the spatial priority map are adjusted such that the high-probability distractor location is inhibited. Without such a learned bias, irrelevant singletons in a search display reduce the gain for input at the target location (Theeuwes & Chen, 2005; Theeuwes et al., 2004), but this distractor cost is less pronounced, and in the current study even absent, when the distractor singleton is presented at the high-probability location. Critically, we found that input gain was also reduced for targets appearing at high-probability distractor locations, providing unequivocal evidence in support of learned suppression operating on a pre-selective stage of priority computation.

In addition to the effects of statistical learning, our findings also confirm that attentional selection of an object is accompanied by the suppression of stimuli in close proximity to the selected object (Caputo & Guerra, 1998; Mounts, 2000a; Mounts, 2000b). Consistent with Theeuwes et al. (2004) the reduced sensitivity at the target location as a function of distractor presence was solely driven by distractors presented near the target and not by more distant distractors. Given our display configurations, distractors and targets that were separated by more than two neutral items had a higher chance of being presented in different than in separate hemifields and thus were less likely to share receptive fields higher up in the processing hierarchy (Sereno & Kosslyn, 1991; Torralbo & Beck, 2008). It has been argued that, to alleviate potential ambiguities in perceptual coding, an inhibitory surround accompanies the selected object that attenuates competition from neighboring objects (Luck, Girelli, McDermott, & Ford, 1997; Mounts, 2000a; Tsotsos, Culhane, Wai, Lai, Davis, & Nuflo, 1995). Alternatively, the target–distractor distance effect could also be explained by a saliency account (Theeuwes, 2004), in which an element surrounded by a homogeneous local environment (as is the case with distant distractors) is more salient than when two singletons are presented nearby, rendering the local environment less homogeneous. According to Nothdurft (1993) saliency depends on local feature contrast, which refers to how different an item is from nearby surrounding items.

Irrespective of the underlying mechanism, however, the current findings suggest that the effect of target–distractor distance is independent from the effect of lingering biases due to statistical learning. Although one should always be careful when interpreting a null finding, as visualized in Figures 2B and 3B, reduced sensitivity for targets with nearby distractors was additive to rather than interacting with learned proactive suppression at the high-probability distractor location. Although it should be noted that our design may have been underpowered to detect an interaction and this finding thus warrants further confirmation, additivity in this case arguably indicates that these two attention factors independently affect two distinct selection stages (Sternberg, 1969). Whereas statistical learning proactively shapes the priority map, the upcoming display configuration additionally shapes the priority gain at different locations, and the combination of these priority landscapes ultimately shapes the efficiency of attentional selection.

It is important to realize that, in the current experiment, participants were not able to make a saccadic eye movement. Indeed, the display was only presented for 100 ms, which is too short to make an directed eye movement (Heeman, Van der Stigchel, Munoz, & Theeuwes, 2019). Nevertheless, it could be argued that fixation position was shifted away from the high-probability distractor location in anticipation of search display onset. Although we cannot indefinitely rule out an alternative explanation in terms of overt shifts of attention, we believe this to be unlikely, as previous work has already demonstrated that statistical distractor learning is robust when fixation is experimentally controlled via an eye tracker (van Moorselaar et al., 2021; Wang & Theeuwes, 2018a; Wang & Theeuwes, 2018b). Moreover, control analyses in the review process showed that the reported distance effect was not exclusive to targets and distractors at the high-probability location but was evident across all display positions. All in all, we are therefore confident that the suppression effect due to statistical learning is purely attentional and not confounded by any eye movement artifacts.

## Supplementary Material

Supplement 1

## Footnotes

1 Due to frequent experiment crashes resulting from memory problems in version 1.3.11, we updated the OSWEB version during data collection.

2 The high-probability location was assigned on basis of the subject identifier code as determined by Qualtrics. The final dataset had on average six observations per display location (range, three to eight).

3 As in our previous work, search times analyses were limited to data of correct trials only. RTs were filtered in a two-step trimming procedure. Trials with RTs shorter than 200 ms or longer than 2000 ms were excluded, after which data were trimmed on the basis of a cutoff value of 2.5 SD from the mean per participant.
