# Supplementary material for: Statistical distractor learning modulates perceptual sensitivity
Source: J Vis. 2021 Nov 5;21(12):3. doi: 10.1167/jov.21.12.3 (PMC8572433; doi:10.1167/jov.21.12.3)
Supplement: Supplement 1 [file jovi-21-12-3_s001.pdf]

Appendix A. Neutral line element control analyses

As visualized in Figure 1A/B, target sensitivity was reliably higher when the line elements inside the target and the neutral element were compatible (i.e., shared the same orientation; Main effect Compatibility: F (1, 47) = 164.6, p < 0.001, n_p^2 = 0.78), indicating that the neutral line element also captured attention (Schreij et al., 2008). Critically, however, our main effect of interest, reduced sensitivity at high probability distractor locations was not modulated by the compatibility effect (F (1, 47) = 0.8, p = 0.037, n_p^2 = 0.017, BF_{01} = 3.7). Consistent with suppression at the high probability distractor location however, the compatibility effect was reliably attenuated when neutral line elements appeared at the high vs. low probability distractor locations (F (1, 47) = 14.5, p < 0.001, n_p^2 = 0.061).

Given that the neutral line element captured attention, in an exploratory analysis we further examined whether the reduced target sensitivity at high probability distractor locations was modulated by the distance between the line elements in the display. As visualized in Figure 1C, if anything, reduced sensitivity at high probability distractor locations was driven by displays where the line elements were further apart. While this is perfectly in line with suppression at the high probability location, which will make the other line element more salient and thus more likely to capture attention (hence leaving insufficient time to redirect attention to the target), neither the main effect of Distance (F (3, 141) = 2.4, p = 0.07, n_p^2 = 0.049, BF_{01} = 8.2) nor the interaction were significant (F (3, 141) = 2.0, p = 0.12, n_p^2 = 0.041, BF_{01} = 38.9).

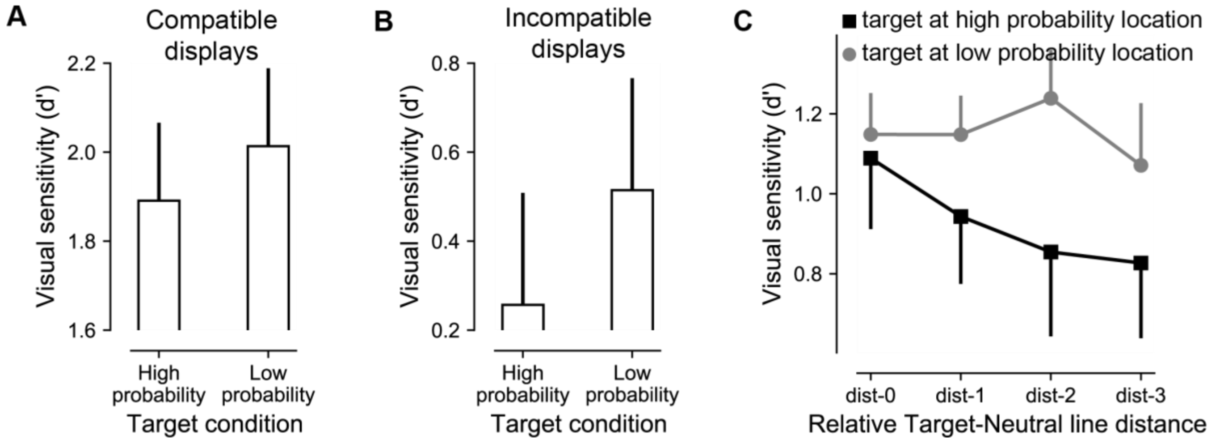

Figure 1 | **A)** *D*' as a function of target condition in compatible displays (i.e., the line elements in the search display share the same orientation). **B)** *D*' as a function of target condition in incompatible displays (i.e., the line elements in the search display have opposite orientations). **C)** *D*' for targets at high and low probability distractor locations as a function of the distance between the two line elements in the display. Note that as in the main paper, in all analyses trials with distractors at high probability distractor locations are excluded.

Appendix B. Eye movement control analysis

As the experiment was conducted online we had no control over subject's eye movements and it is thus unclear whether participants adhered to the fixation instructions. Although we cannot indefinitely rule out an alternative explanation in terms of overt shifts of attention several aspects of the data indicate that our results cannot be explained by such an alternative account. First, if participants indeed shifted fixation away from the high probability distractor location, one would expect a pronounced gradient of target enhancement surrounding the position *opposite* to the high probability distractor location. As visualized in Figure 2A, however, this was clearly not the case. Indeed, a repeated measures ANOVA with within subjects' factor gradient (pos1, pos2, pos3, pos4) excluding the high probability distractor position (i.e., pos0) did not yield a reliable effect (F (2, 107) = 0.8, p = 0.47). Second, if the linear trend at high probability locations observed in the distance analyses (Figures 2B/3B in the main paper) should be attributed to a (strategic) overt shift of attention away from the high probability location, this pattern should be exclusive for targets at the high probability location. However, as visualized in Figure 2B, the reported distance effect, as also reported in Theeuwes et al. (2004), was not exclusive to targets/distractors at the high probability location, but was evident across all display positions.

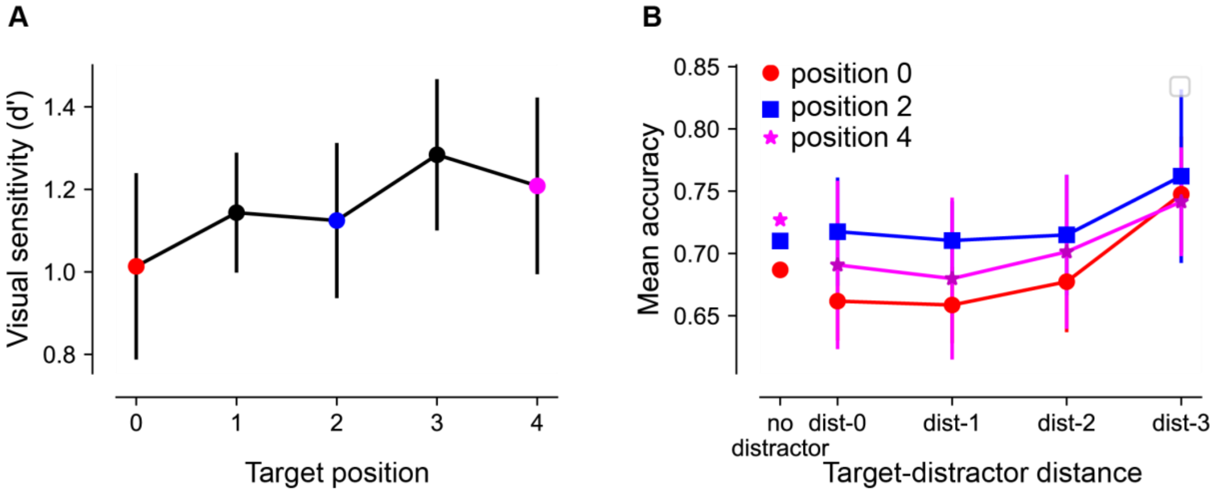

Figure 2 | **A)** *D*' as a function of target position relative to the high probability distractor location. Position 0 means the high probability distractor location; position 1 means the low probability distractor location with one unit (45° polar angle) away from the high probability distractor location, and so on. Data only includes distractor absent displays. **B)** Mean accuracy for Target positions 0, 2 and 4 as a function of target-distractor distance.
